# Nanobody-Dependent Delocalization of Endocytic Machinery in *Arabidopsis* Root Cells Dampens Their Internalization Capacity

**DOI:** 10.3389/fpls.2021.538580

**Published:** 2021-03-19

**Authors:** Joanna Winkler, Andreas De Meyer, Evelien Mylle, Veronique Storme, Peter Grones, Daniël Van Damme

**Affiliations:** ^1^Department of Plant Biotechnology and Bioinformatics, Ghent University, Ghent, Belgium; ^2^VIB Center for Plant Systems Biology, Ghent, Belgium

**Keywords:** nanobody, endocytosis, *Arabidopsis*, protein delocalization, fluorescence microscopy, TPLATE complex (TPC)

## Abstract

Plant cells perceive and adapt to an ever-changing environment by modifying their plasma membrane (PM) proteome. Whereas secretion deposits new integral membrane proteins, internalization by endocytosis removes membrane proteins and associated ligands, largely with the aid of adaptor protein (AP) complexes and the scaffolding molecule clathrin. Two AP complexes function in clathrin-mediated endocytosis at the PM in plant cells, the heterotetrameric AP-2 complex and the hetero-octameric TPLATE complex (TPC). Whereas single subunit mutants in AP-2 develop into viable plants, genetic mutation of a single TPC subunit causes fully penetrant male sterility and silencing single subunits leads to seedling lethality. To address TPC function in somatic root cells, while minimizing indirect effects on plant growth, we employed nanobody-dependent delocalization of a functional, GFP-tagged TPC subunit, TML, in its respective homozygous genetic mutant background. In order to decrease the amount of functional TPC at the PM, we targeted our nanobody construct to the mitochondria and fused it to TagBFP2 to visualize it independently of its bait. We furthermore limited the effect of our delocalization to those tissues that are easily accessible for live-cell imaging by expressing it from the PIN2 promoter, which is active in root epidermal and cortex cells. With this approach, we successfully delocalized TML from the PM. Moreover, we also show co-recruitment of TML-GFP and AP2A1-TagRFP to the mitochondria, suggesting that our approach delocalized complexes, rather than individual adaptor complex subunits. In line with the specific expression domain, we only observed minor effects on root growth, yet realized a clear reduction of endocytic flux in epidermal root cells. Nanobody-dependent delocalization in plants, here exemplified using a TPC subunit, has the potential to be widely applicable to achieve specific loss-of-function analysis of otherwise lethal mutants.

## Introduction

Cells are delineated by their plasma membrane (PM). The PM houses a plethora of proteins ranging from receptors and ion channels to structural membrane proteins. Many of these PM proteins, commonly termed cargo, are responsible for cellular communication with the outside world. In eukaryotes, endocytosis is the cellular process where cargoes, associated ligands as well as lipids are internalized from the PM. Endocytosis thereby provides a way to regulate the content and consequently modulate protein activity at the PM. A predominant and well-studied form of endocytosis is clathrin-mediated endocytosis (CME; Bitsikas et al., 2014). CME refers to the dependency of the scaffolding protein clathrin, which coats the developing and mature vesicles (Robinson, 2015). In plants, CME plays a role in hormone signaling (Irani et al., 2012; Martins et al., 2015; Zhang et al., 2017), nutrient availability (Wang et al., 2017; Dubeaux et al., 2018; Yoshinari et al., 2019), pathogen defense and susceptibility (Mbengue et al., 2016; Li and Pan, 2017), and other biotic and abiotic stresses (Li et al., 2011). Consequently, CME is essential for plant development.

Two early-arriving adaptor complexes, the heterotetrameric Adaptor Protein-2 complex (AP-2) and the hetero-octameric TPLATE complex (TPC) facilitate CME in plants (Gadeyne et al., 2014). In contrast to AP-2, TPC represents an evolutionary ancient protein complex, which is lost in yeast and mammalian cells (Hirst et al., 2014). The slime mold *Dictyostelium discoideum* contains a similar complex, named TSET. TSET, however, is a hexameric complex in contrast to TPC in *Arabidopsis thaliana*, which has two additional subunits. Also contrary to TPC, TSET is dispensable in *D. discoideum* (Hirst et al., 2014). The presence of a full or partial TSET complex in other eukaryotes was confirmed by additional homology searches, indicative of its ancient evolutionary origin (Hirst et al., 2014).

Adaptor Protein-2 complex and TPC have both common and distinct functions, possibly relating to cargo specificity and/or fate of the internalized cargo (Bashline et al., 2015; Sánchez-Rodríguez et al., 2018; Wang et al., 2019; Yoshinari et al., 2019). In addition, functional diversification of both complexes is reflected in their mutant phenotypes. Knockout plants in individual AP-2 subunits are affected at various stages of development but viable (Bashline et al., 2013; Di Rubbo et al., 2013; Fan et al., 2013; Kim et al., 2013; Yamaoka et al., 2013). However, *ap2* mutants show reduced internalization of the styryl dye N-(3-Triethylammoniumpropyl)-4-(6-(4-(Diethylamino) Phenyl) Hexatrienyl) Pyridinium Dibromide (FM4-64), which can be seen as proxy to a difference in cargo uptake (Jelínková et al., 2010), as well as known endocytic cargoes like the brassinosteroid receptor BRASSINOSTEROID INSENSITIVE 1 (BRI1), the boron exporter BOR1, and auxin efflux carriers of the PIN-FORMED protein family (Di Rubbo et al., 2013; Fan et al., 2013; Kim et al., 2013; Yoshinari et al., 2016, 2019).

The relatively mild phenotype of *ap2* single subunit mutants in plants contrasts with the lethal phenotype of a single *ap2* subunit knockout in mice (Mitsunari et al., 2005). Alternatively, the complex does not seem to be essential for yeast (Yeung et al., 2013). In *Caenorhabditis elegans*, AP-2 subunits are capable of assembling into hemicomplexes which partially retain their functionality (Gu et al., 2013). In plants, AP2M and AP2S are still recruited to the PM in *ap2s* and *ap2m* mutants, respectively (Wang et al., 2016), suggesting that AP-2 hemicomplexes might also confer partial functionality in plants.

In contrast to AP-2, single knockouts of TPC subunits result in fully penetrant male sterility with shriveled pollen and ectopic callose accumulation (Gadeyne et al., 2014). Similar pollen-lethal phenotypes are also reported for a mutant in DYNAMIN-RELATED PROTEIN 1C protein (*drp1c*; Backues et al., 2010), as well as a CLATHRIN LIGHT CHAIN mutant, *clc1* (Wang et al., 2013), involved in vesicle fission and clathrin triskelion assembly, respectively.

So far, there is only one viable weak allele of one TPC subunit identified. This *twd40-2-3* mutant (Bashline et al., 2015) is however likely merely a knockdown as *twd40-2-1* and *twd40-2-2* mutants are pollen lethal (Gadeyne et al., 2014). Knockdowns of *TML* and *TPLATE* resulted in seedling lethality with a reduced internalization of FM4-64, BRI1, RECEPTOR-LIKE PROTEIN 44 (RLP44), and the cellulose synthase subunit CESA6 (Irani et al., 2012; Gadeyne et al., 2014; Sánchez-Rodríguez et al., 2018; Gómez et al., 2019). Silencing works on the messenger level and phenotypes only become apparent following degradation of pre-made proteins. As AP complexes can be recycled following each round of internalization, approaches affecting these complexes at the protein level have a more direct effect. In animal cells, conditional delocalization using rapamycin to target AP-2 to mitochondria has been successfully applied to interfere with endocytosis (Robinson et al., 2010). Also, epidermal growth factor receptor substrate 15 (EPS15), a pioneer endocytic accessory protein, was successfully inactivated in HeLa cells by expressing an anti-EPS15 nanobody on endosomes or mitochondria (Traub, 2019).

Since their discovery, nanobodies, derived from camelid heavy chain-only antibodies (HCAb), have found their way into a wide variety of applications in biological fields. Nanobodies are similar to antibodies (Ab) in the sense that they can bind epitopes with high affinity in a highly selective manner (Ingram et al., 2018). Their applications range from drug discovery, crystallography, and imaging techniques to probing protein functions by degradation or delocalization (Caussinus et al., 2012; Früholz et al., 2018; Ingram et al., 2018). Nanobodies can be expressed as a single chain, compact and stable protein while still retaining high selectivity and affinity for its epitope (Muyldermans, 2013). This makes them more convenient to clone and to express compared to conventional Ab.

In plants, nanobodies have been used to selectively degrade proteins using the deGradFP method, originally developed in *Drosophila melanogaster*. DeGradFP links an anti-GFP nanobody to an F-box protein, thereby targeting it for ubiquitin-dependent degradation (Caussinus et al., 2012). This approach was shown to be functional in plants (Baudisch et al., 2018) and successfully used to deplete WUSCHEL-GFP in the *Arabidopsis* flowering meristem (Ma et al., 2019) and the centromeric Histon H3 of *Arabidopsis* in transgenic tobacco plants (Sorge et al., 2021). Nanobodies have also been used in *Arabidopsis* seedlings to lock down vacuolar sorting receptors (VSRs) in cellular compartments upstream of trans-Golgi network/early endosomes, allowing to determine their retrograde trafficking pathway (Früholz et al., 2018).

Here, we explore, similar to what has been done in animal cells (Robinson et al., 2010; Traub, 2019), the effects on CME caused by lockdown of the endocytic machinery. We use a GFP-tagged functional TML-GFP fusion protein in the homozygous *tml-1(−/−)* mutant background and delocalized it to the mitochondria using a nanobody directed against eGFP.

## Materials and Methods

### Cloning

Gateway entry clones pDONR221-TagBFP2, pDONR221-MITOTagBFP2 and pDONRP2RP3-GFPNb were generated according to the manufacturer’s instructions (ThermoFisher Scientific BP clonase). pDONR221-TagBFP2 was amplified from pSN.5 mTagBFP2 (Pasin et al., 2014) with primers:

AttB1-GGGGACAAGTTTGTACAAAAAAGCAGGCTATGTCATCTAAGGGTGAAGAGC TTATC AAAGAGAAT and AttB2-GGGGACCACTTTGTACAAGAAAGCTGGGTCACCTCCGCCACCTCCACCTCCCAGTCCTGCGTA.

pDONR221-MITOTagBFP2 was generated from pDONR221-TagBFP2 by including the import signal of the yeast mitochondrial outer membrane protein Tom70p as described before (Robinson et al., 2010). The following primers sequences were used:

AttB1-GGGGACAAGTTTGTACAAAAAAGCAGGCTCAATGAAGAGCTTCATTACAAGGAACAAGACAGCCATTTTGGC AACCGTTGCTGCTACAGGTACTGCCATCGGTGCCTACTATTATTACAACCAATTGCAACAGGATCCACCGGTCGCCACCATGTCATCTAAGGGTGAAGAGCTT and AttB2-GGGGACCACTTTGTACAAGAAAGCTGGGTACGCTAAGTCTTCCTCTGAAATCAA.

pDONRP2RP3-GFPNb was generated from an anti-GFP Nanobody construct (Künzl et al., 2016) with primers attB2-GGGGACAGCT TTCTTGTACAAAG TGGGGATGTATCCTTA TGATGTTC and attB3r-GGGGACAACTT TGTATAATAAAGT TGTTTAAT GATGATGATGA TGATGAGAAGA including a HA-tag, a 3xHis-tag, and a stop codon.

The entry clones of the PIN2 promoter pDONRP4P1R_PIN2prom (Marquès-Bueno et al., 2016) or 35 s promoter, pDONR221-MITOTagBFP2 and pDONRP2RP3-GFPNb were used in a triple Gateway LR reaction, combining pB7m34GW (Karimi et al., 2005) to yield pB7m34GW_PIN2prom::MITOTagBFP2-GFPNb or pB7m34GW_p35sprom::MITOTagBFP2-GFPNb.

### *Nicotiana benthamiana* Plant Growth and Transient Expression Assay

*Nicotiana benthamiana* plants were grown in a greenhouse under long-day conditions (6–22 h light, 100 PAR, 21°C) in soil (Saniflo Osmocote pro NPK: 16 − 11 − 10+ magnesium and trace elements). Transient expression was performed by leaf infiltration according to (Sparkes et al., 2006). The abaxial epidermis was imaged 48 h after infiltration.

### *Arabidopsis* Plant Material and Transformation

Plants expressing pB7m34GW_PIN2prom::MITOTagBFP2-GFPNb were generated by floral dip (Clough and Bent, 1998). Constructs were dipped into Col-0 and *tml-1(−/−)* (At5g57460) mutant lines described previously (Gadeyne et al., 2014). Primary transformants (T1) were selected on BASTA containing ½ strength MS medium without sucrose and 0.6% Gelrite (Duchefa, Netherlands). PIN2prom::MITOTagBFP2-GFPNb expression was analyzed in the progeny of BASTA-resistant primary transformants (T2 seeds) by microscopy and T2 lines demonstrating strong expression were selected regardless of insert copy number. Next, T2 lines were crossed with the previously described TML-GFP complemented *tml-1(−/−)* mutant line expressing also RPS5Aprom::AP2A1-TagRFP (Gadeyne et al., 2014). Primary hybrids were analyzed *via* microscopy and best lines were selected on the basis of both PIN2prom::MITOTagBFP2-GFPNb and RPS5Aprom::AP2A1-TagRFP expression. For both Col-0 and *tml-1(−/−)* backgrounds, two independent lines (-Nb1 and -Nb2) were generated. Namely, Col-Nb1, Col-Nb2, TML-Nb1, and TML-Nb2. In order to synchronize the age and overall fitness of the seeds, all lines used in the root growth and carbon starvation study, including the Col-0 and *tml-1(−/−)* lines, were propagated together and collected at the same time.

### Quantification of TML-GFP Endocytic Foci

Four to five days old seedlings of TML, TML-Nb1, and TML-Nb2 were grown on ½ strength MS medium without sucrose and 0.6% Gelrite (Duchefa, Netherlands). Acquired pictures were analyzed in Fiji/ImageJ (Schindelin et al., 2012; Schneider et al., 2012). In each analyzed root an ROI of constant dimensions was selected. In addition in TML-Nb1 and TML-Nb2, the ROI did not overlap with mitochondrial clusters. The median intensity in each root was recorded, followed by the counts of the endocytic foci using the Find Maxima function. Statistical differences in median intensity and foci count between the lines were analyzed with pairwise Wilcoxon tests. *p*-values were adjusted using the Bonferroni method.

### Phenotypical Quantification of Root Growth

*Arabidopsis* seedlings were grown on ½ strength MS medium without sucrose and 0.6% Gelrite (Duchefa, Netherlands). Seeds of all lines were equally placed on the plates (3 seeds per line per plate, distributed over 14 plates). Plates were left for the stratification for 48 h at 4°C, and then placed at 21°C in continuous light. Two days after transfer to the light, seeds which did not germinate were marked and excluded in the further analysis. For root growth analysis, seedlings were grown in continuous light and 2 days after germination the root growth of every seedling was marked for 7 days, daily. For carbon starvation, seedlings were grown for 5 days, including the germination period, in continuous light after which the root growth of every seedling was marked. Subsequently, the plates were covered with aluminum foil and left for 7 days in dark after which root growth was marked again. Root growth and carbon starvation assays measurements were carried out with Fiji/ImageJ (Schindelin et al., 2012; Schneider et al., 2012). Seedlings, which stopped growing at early time points of the study, have grown into the medium or have grown in direct contact with the plate edge, were excluded from further analysis. Statistical differences for root growth assays were determined *via* a mixed model analysis. Mixed linear model analysis was applied to the root length of the lines Col-0, Col-Nb1, Col-Nb2, TML, TML-Nb1, and TML-Nb2 using the mixed procedure from SAS (SAS Studio 3.8 and SAS 9.4, SAS Institute Inc., Cary, NC). Fixed effects in the model were Line, Day, and the interaction term. An unstructured covariance structure was estimated to model the correlations between measurements done on the same plant. The degrees of freedom of the fixed effects were approximated with the Kenward-Rogers method. The hypotheses of interests were the differences between Col-0 and its respective nanobody lines, between TML and its respective nanobody lines and between the nanobody lines with the same background. These hypotheses were tested using the plm procedure. *p*-values were adjusted for multiple testing using the maxT procedure as implemented in the plm procedure. For the carbon starvation assay, statistical differences in root growth between the lines were analyzed with pairwise Wilcoxon tests. *p*-values were adjusted using the Bonferroni method.

### FM-Uptake Quantification

Endocytic tracer FM4-64 stock solution was prepared prior to treatment (2 mM in DMSO, Thermo Fisher). Roots were stained with 2 μM FM4-64 by incubating the seedlings in FM-containing ½ strength MS medium without sucrose for 30 min. Treatment was followed by microscopy. Acquired pictures were analyzed in Fiji/ImageJ (Schindelin et al., 2012; Schneider et al., 2012). PM and cytosol of individual epidermal cells were outlined (using the Select Brush Tool and Freehand selections, respectively) and histograms of pixel intensities were generated. Pictures which contained more than 1% saturated pixels were excluded from the quantification. Cytoplasm/PM ratios were calculated from average intensities of the top 1% highest intensity pixels based on the histograms. Outliers were removed *via* interquartile range (IQR, data point ruled out if its value was either lower than first quartile Q1-1.5 × IQR, or higher than third quartile Q3-1.5 × IQR) in a single step. The normality assumption of the measurements was verified with the Shapiro-Wilk normality test. Due to violation of the normality assumption, statistical differences in intensity values between the lines were analyzed with pairwise Wilcoxon tests. *p*-values were adjusted using the Bonferroni method. All Shapiro-Wilk normality tests and Wilcoxon tests were performed using the R statistical software. (Rstudio Team, 2019, Version 1.2.5033, R Version 3.6.2).

### Image Acquisition

**Figure 1:**
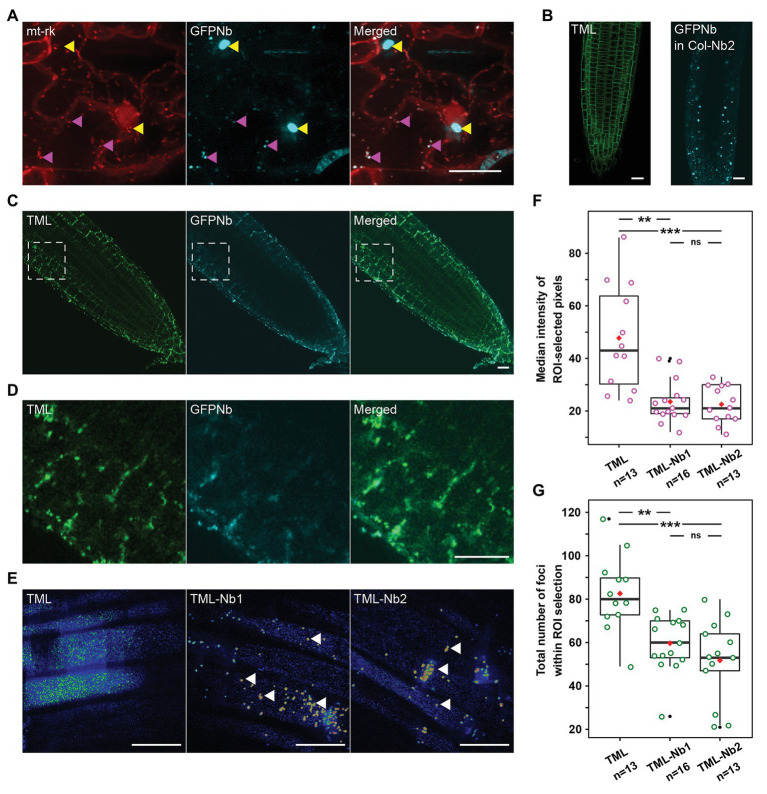
Expression of a mitochondrial-targeted nanobody against GFP allows delocalization of TML-GFP. **(A)** Representative Z-stack projection of epidermal *Nicotiana benthamiana* cells transiently expressing the mitochondrial marker mt-rk (red) and the 35S::MITO-TagBFP2-GFP-Nanobody (GFPNb, cyan). Both fusion proteins colocalize at small cytosolic punctae (magenta arrowheads), but not at big aggregates, likely representing dysfunctional clustered mitochondria (yellow arrowheads). **(B)** Representative *Arabidopsis* root image of *tml-1(−/−)* complemented with TML-GFP showing that the functional TML fusion is predominantly targeted to the PM (left) as well as localization of GFPNb in cytosolic punctae in the WT (Col-0) background (right). **(C,D)** Representative overview images and respective zooms of the outlined region of *Arabidopsis* roots where TML-GFP in *tml-1(−/−)* was combined with MITOTagBFP2-GFPNb expression, leading to its delocalization from the PM. **(E)** Representative, rainbow intensity colored, grazing sections through the PM, showing the recruitment of TML to endocytic foci without (left) and with partial delocalization of TML-Nb1 and TML-Nb2 (middle, right, white arrowheads). Scale bars equal 20 μm. **(F,G)** Box plots showing the median intensity **(F)** or total number **(G)** of endosomal TML-GFP positive foci in the *Arabidopsis* roots. In the roots expressing GFPNb, both the intensity of the foci as well as their number are significantly reduced compared to TML-GFP (Wilcoxon pairwise comparison tests with Bonferroni adjusted *p*-values. *p* < 0.001 are represented as ***, <0.01 as **, and non-significant values as “ns”). The black lines represent the median and the red diamonds represent the mean of the analyzed values. Each magenta or green dot represents an individual cell. Black dot refers to outliers. *n* refers to the total number of analyzed cells.

*Nicotiana benthamiana* infiltration assay imaging (Figure 1A) was performed on a PerkinElmer UltraView spinning-disk system, attached to a Nikon Ti inverted microscope and operated using the Volocity software package. Images were acquired on an ImagEMccd camera (Hamamatsu C9100-13) using frame-sequential imaging with a 60x water immersion objective (NA = 1.20). Specific excitation and emission was performed using a 405 nm laser excitation combined with a single band pass filter (454–496 nm) for TagBFP2 and 561 nm laser excitation combined with a dual band pass filter (500–530 and 570–625 nm) for RFP. Images shown are Z-stack projections. Z-stacks were acquired in sequential frame mode with a 1 μm interval using the UltraView (Piezo) focus drive module.

**Figure 2:**
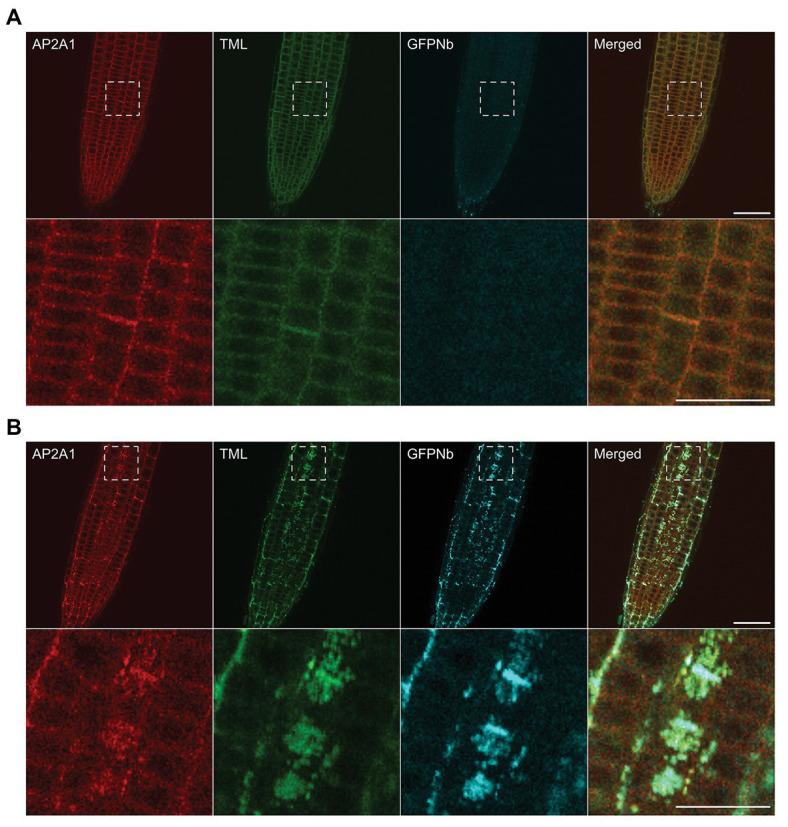
Delocalization of TML also affects the targeting of other endocytic players. **(A,B)** Representative images and blow-ups of the outlined regions of *Arabidopsis* roots expressing TML-GFP and AP2A1-TagRFP without **(A)** and with **(B)** MITOTagBFP2-GFPNb expression. GFPNb expression causes delocalization of both TML and AP2A1. Scale bars equal 20 μm (overview pictures) or 10 μm (blow-up pictures).

Confocal *Arabidopsis* images were taken using Leica SP8X confocal microscope equipped with a White Light Laser and using the LASX software (Figures 1B-D, 2, 4). Images were acquired on Hybrid (HyD, gating 0.3–10.08 ns) and Photomultiplier (PMT) Detectors using bidirectional line-sequential imaging with a 40x water objective (NA = 1.10) and frame or line signal averaging. Specific excitation and emission were used: 405 nm laser and filter range 410–470 nm for TagBFP2, 488 nm laser and filter range 500–550 nm for GFP, 488 nm laser, and filter range 600–740 nm for FM4-64, 555nm laser and filter range 560–670 for TagRFP. Focal planes of PMs (Figure 1E) were acquired with the PerkinElmer UltraView spinning-disk system using a 100x oil immersion objective (NA = 1.45). Specific excitation and emission was performed using a 488 nm laser combined with a single band pass filter (500–550 nm) for GFP. Images shown are single-slice.

**Figure 3:**
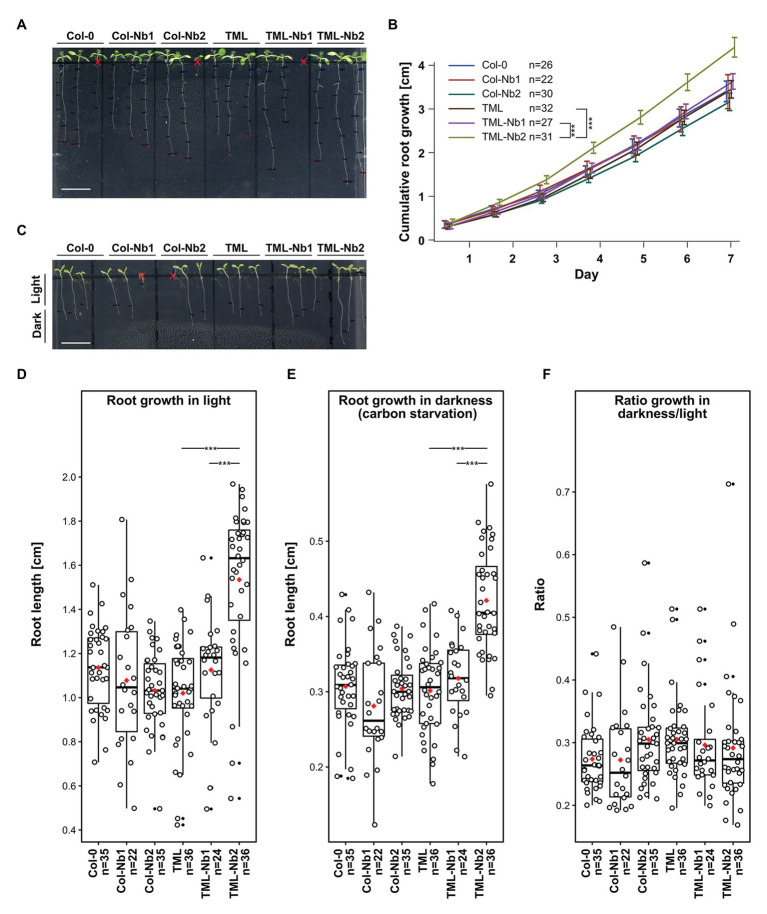
Delocalizing TML-GFP in root epidermal and cortical cells does not adversely affect root growth. Comparison of wild type seedlings (Col-0), wild type seedlings expressing MITOTagBFP2-GFPNb (Col-Nb1 and Col-Nb2) complemented *tml-1(−/−)* mutants expressing TML-GFP (TML) and complemented *tml-1(−/−)* mutants expressing TML-GFP and MITOTagBFP2-GFPNb (TML-Nb1 and TML-Nb2) in different light conditions. **(A,B)** Representative images of seedlings and quantification of root growth in continuous light. There are no statistical differences between the lines except TML-Nb2 which showed enhanced cumulative root growth. **(C-F)** Representative images of seedlings grown for 5 days in continuous light and subsequently for 7 days in continuous dark. Consistent with the cumulative root growth assay, the TML-Nb2 grew bigger compared to the TML-GFP and TML-Nb1 under both growth conditions **(D,E)**. However, there were no significant differences in the analysis of the ratio of darkness/light growth **(F)**. Scale bars in A and C equal 1 cm. Red crosses in A and C are marking seedlings that were excluded from the analysis due to delayed germination or due to roots growing in the agar. The measurements of growth in light, dark, as well as the respective dark/light ratio, are represented as jitter box plots. The black lines represent the median and the red diamonds represent the mean of the analyzed values. Each dot represents an individual cell and black dost refer to outliers. *n* refers to the total number of analyzed cells. Significant statistical differences in growth between the lines, based on Wilcoxon pairwise comparisons tests are indicated. *p* < 0.001 are represented as *** (Bonferroni adjusted *p*-values).

## Results

### The Targeting Sequence of Yeast Tom70p Targets the Nanobody to the Mitochondria

We expressed a nanobody directed against eGFP (GFPNb; Künzl et al., 2016), which we visualized by fusing it to TagBFP2. We targeted the fusion protein to the mitochondria using the import signal of the yeast mitochondrial outer membrane protein Tom70p as described before (Robinson et al., 2010). This targeting signal is functional in plants as constructs containing this signal colocalized with MitoTracker in *N. benthamiana* leaf epidermal cells (Winkler et al., 2021). To verify that the GFP nanobody did not interfere with the mitochondrial targeting, we expressed it under the control of the 35Sprom together with the red mitochondrial mt-rk marker (Nelson et al., 2007) in *N. benthamiana* abaxial leaf epidermal cells and visualized both fusion proteins using spinning disk confocal microscopy. Both constructs colocalized at discrete punctae, but not exclusively. We observed that the mt-rk marker did not localize to big aggregates labeled with the GFPNb (Figure 1A). The mt-rk marker line consists of yeast cytochrome c oxidase IV (Nelson et al., 2007). It has been shown that release of cytochrome c oxidase into the cytosol is associated with changes of mitochondrial integrity (Kadenbach et al., 2004). It is, therefore, possible that the observed signal from aggregates originates from mitochondrial clustering similarly to what we observed with our knocksideways in plants system (Winkler et al., 2021). The absence of the mt-rk fusion protein from those clusters might therefore be a consequence of altered fitness of the clustered mitochondria.

### A Mitochondrially Targeted Nanobody Can Delocalize TML

TPLATE complex is a robust multi-subunit complex functioning at the PM and can be affinity purified using any of its subunits as bait (Gadeyne et al., 2014). In order to delocalize, and thereby inactivate TPC, we took advantage of the functionally complemented homozygous *tml-1(−/−)* mutant expressing TMLprom::TML-GFP (Gadeyne et al., 2014). In complemented *tml-1(−/−) Arabidopsis* roots, TML-GFP is recruited predominantly at the PM (Figure 1B, left). We introduced our MITO-TagBFP2-GFPNb nanobody into this background and used PIN2prom to drive expression of the construct. PIN2prom expresses in epidermis and cortex root cell files, which, with respect to future experiments, would allow us easily to perform confocal microscopy. Two independent lines, TML-Nb1 and TML-Nb2, were selected. MITO-TagBFP2-GFPNb (GFPNb) analogous to *N. benthamiana* leaves, localized to discrete punctae also in *Arabidopsis* wild type roots (Figure 1B, right).

Co-expression with GFPNb changed the uniform PM labeling of TML to a denser staining of discrete punctae in epidermis and cortex. Most of those were still near the PM and colocalized with the fluorescent signal from the nanobody, indicating effective delocalization of TML-GFP (Figure 1C and enhanced in Figure 1D). This delocalization was not apparent in the deeper layers of the root, where TML remained uniformly recruited to the PM (Figure 1C). Detailed analysis using spinning disk confocal microscopy confirmed the strong recruitment of TML to mitochondria that were present in the focal plane of the PM (Figure 1E, arrowheads). Next to the mitochondria, however, TML remained recruited to endocytic foci at the PM in root epidermal cells. The density of endocytic foci in epidermal root cells is very high (Dejonghe et al., 2016, 2019; Sánchez-Rodríguez et al., 2018). The density and intensity of the endocytic foci, marked by TML-GFP, appeared higher in epidermal cells in the complemented mutant (control) compared to the two independent lines expressing the GFPNb. Quantification showed a marked decrease in median signal intensity at the PM in the GFPNb expressing lines in regions devoid of mitochondria. The lower intensity of the signal also led to a statistically reduced number of foci (maxima) that could be detected. The reduced median intensity and lower amount of foci detected are in agreement with a substantial amount of TML-GFP accumulating at the mitochondria (Figures 1F,G).

### Nanobody-Dependent Delocalization of TML Also Affects Other Endocytic Players

In plants, the heterotetrameric AP-2 complex and the octameric TPC are presumed to function largely, but not exclusively, together to execute CME (Gadeyne et al., 2014; Bashline et al., 2015; Wang et al., 2016; Adamowski et al., 2018). Both TPC and AP-2 have been shown to be involved in the internalization of cellulose synthase (CESA) complexes or the brassinosteroid receptor BRI1 for example (Bashline et al., 2013, 2015; Di Rubbo et al., 2013; Gadeyne et al., 2014; Sánchez-Rodríguez et al., 2018).

Moreover, a joint function is also suggested from proteomics analyses, which could identify subunits of both complexes when the AtEH1/Pan1 TPC subunit was used as bait in tandem-affinity purification assays (Gadeyne et al., 2014). To investigate whether our tool, aimed at delocalizing TPC, would also interfere with AP-2 recruitment at the PM, we tested the localization of AP-2 when TML was targeted to the mitochondria. To do so, we crossed our TML-GFP line, in *tml-1(−/−)* and expressing PIN2prom::MITOTagBFP2-GFPNb with the homozygous complemented *tml-1(−/−)* line, expressing TML-GFP as well as one of the large AP-2 subunits, AP2A1, fused to TagRFP (Gadeyne et al., 2014). Offspring plants that did not inherit the nanobody construct showed PM and cell plate recruitment of TML and AP2A1, and only background fluorescence in the TagBFP2 channel (Figure 2A). In the offspring plants that inherited the nanobody construct, however, the localization of the adaptor complex subunits changed. Both TML and AP2A1 accumulated at punctae, which clearly colocalized with the TagBFP2-fused nanobody construct (Figure 2B). The observed delocalization of AP2A1 to the mitochondria, together with TML strongly suggests that our approach has the capacity to delocalize TPC and AP-2 rather than TML alone, given that TPC and AP-2 are presumed to be linked *via* the AtEH1/Pan1 subunit (Gadeyne et al., 2014).

### Mistargeting Adaptor Complexes in Epidermis and Cortex Affects Root Endocytic Uptake With Only Minor Effects on Root Growth

In contrast to AP-2, genetic interference with TPC subunits causes fully penetrant male sterility (Van Damme et al., 2006; Di Rubbo et al., 2013; Fan et al., 2013; Kim et al., 2013; Yamaoka et al., 2013; Gadeyne et al., 2014). TPC functionality, therefore, requires all subunits, and constitutive homozygous loss-of-function backgrounds are therefore non-existing. Abolishing endocytosis in plants, by silencing TPC subunits (Gadeyne et al., 2014) or overexpression of the uncaging proteins AUXILLIN-LIKE 1 or 2 (Adamowski et al., 2018) severely affects seedling development. The effect of silencing TPC subunits only indirectly affects protein levels and targeting clathrin might interfere with trafficking at endosomes besides the PM. As TPC and AP-2 only function at the PM, inactivating their function should not directly interfere with more downstream aspects of endosomal trafficking. Furthermore, by restricting the expression domain where the adaptor complex function is tuned down to the two outermost layers in the root should allow to study internalization from the PM, independently of possible indirect effects caused by the severe developmental alterations.

We evaluated the growth of several different lines expressing either GFPNb alone: Col-Nb1 and Col-Nb2, or GFPNb combined with TML-GFP in the complemented *tml-1(−/−)* mutant background: TML-Nb1 (−/−) and TML-Nb2 (−/−). At the seedling level, we did not observe any major adverse developmental effects (Figure 3A). Root length measurements of light grown seedlings revealed enhanced growth in the TML-Nb2 line compared to TML and TML-Nb1 (Figure 3B). The observed variability of this line, compared to all other lines that behaved similarly, probably results from a positional effect of the insertion. Nevertheless, our results show that nanobody expression in the PIN2prom domain and partial delocalization of TML has no negative effect on seedling development under normal growth conditions.

**Figure 4:**
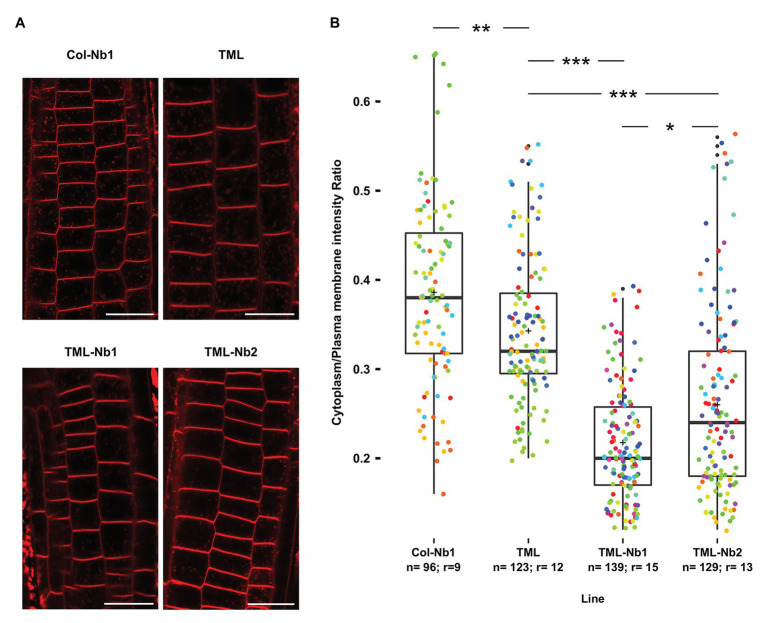
Nanobody-dependent delocalization reduces endocytic flux. **(A)** Representative single confocal slices of FM4-64 stained root cells of the different lines for which endocytic flux was quantified. FM4-64 uptake was compared between wild type *Arabidopsis* expressing MITOTagBFP2-GFPNb (Col-Nb1), the TML-GFP expressing complemented *tml-1(−/−)* mutant (TML), and two independent lines of the TML-GFP expressing complemented *tml-1(−/−)* mutant expressing MITOTagBFP2-GFPNb (TML-Nb1 and TML-Nb2). Scale bars equal 20 μm. **(B)** Jittered box plot representation of the quantification of the cytoplasm/plasma membrane intensity of FM4-64 as proxy for endocytic flux. The black lines represent the median and the crosses represent the mean values. The dots represent individual measurements of cells. The rainbow-colored indication of the dots groups the cells from the different roots that were analyzed. The number of cells (*n*) and the number of individual roots (*r*) are indicated in the graph. The indicated *p*-values were calculated using pairwise Wilcoxon tests and corrected using the Bonferroni method. Significant statistical differences between the lines based on Wilcoxon pairwise comparisons tests are indicated. *p* < 0.001 are represented as ***, < 0.01 are represented as ** and < 0.1 are represented as * (Bonferroni adjusted *p*-values).

The AtEH/Pan1 TPC subunits were recently implicated in growth under nutrient-depleted conditions as downregulation of *AtEH1/Pan1* expression rendered plants hyper-susceptible to carbon starvation (Wang et al., 2019). We, therefore, assessed if delocalizing TML-GFP, as well as other endocytic players, would also render these plants susceptibility to nutrient stress. To do so, we measured root lengths of seedlings grown for 5 days in continuous light and afterwards we placed them in the dark for an additional 7 days. Measurements of root growth in the dark under carbon stress conditions did not show any differences between WT and Col-Nb lines. However, also here, the TML-Nb2 line exhibited increased root growth compared to TML and TML-Nb1 in both light and dark conditions (Figures 3C-E). We calculated the ratio of root growth in dark over root growth in light to avoid overestimation of the results due to the extraordinary growth of TML-Nb2 line. The ratios revealed that sequestering TML in TML-Nb lines did not cause any negative effect as the ratios were similar between all lines tested (Figure 3F). Overall, the effects of TML relocalization did not reveal any defects on seedling development, even under nutrient-stress conditions.

The subtle differences observed by comparing the effect of delocalization of TML on plant growth are likely a consequence of the restricted expression domain of GFPNb. We, therefore, monitored the effects of delocalizing TML more directly by visualizing the internalization of the styryl dye FM4-64, which in plants is commonly used as proxy for endocytic flux (Rigal et al., 2015; Jelínková et al., 2019). To rule out indirect effects of targeting GFPNb to the mitochondria, we compared endocytic flux between Col-Nb1, TML-GFP in *tml-1(−/−)*, TML-Nb1 (−/−), and TML-Nb2 (−/−). We observed a slight decrease in endocytic flux when comparing wild type seedlings with the complemented *tml-1(−/−)* line and a strong reduction in endocytic flux between the complemented mutant and both complemented mutant lines where TML was partially delocalized (Figures 4A,B). Direct visualization of endocytic flux, therefore, allowed us to conclude that expression of the PIN2prom::MITOTagBFP2-GFPNb has the capacity to interfere with endocytosis in *Arabidopsis* root epidermal cells and that this tool certainly has the capacity to generate knockdown, and maybe even knockout lines at the protein level.

## Discussion

Analyzing how impaired TPC function directly affects endocytosis is hampered by the male sterility and/or seedling lethal mutant phenotypes following genetic interference of individual subunits (Gadeyne et al., 2014). Here, we explored to impair TPC function at the protein level by delocalizing a functional and essential subunit in its respective complemented mutant background. We were inspired by previous work in animal cells. However, instead of using rapamycin-dependent rerouting of one of the large AP-2 subunits, combined with silencing the endogenous subunit (Robinson et al., 2010), we took advantage of the complemented *tml-1(−/−)* mutant line expressing TML-GFP (Gadeyne et al., 2014) in combination with targeting a nanobody directed against GFP (GFPNb; Künzl et al., 2016) to the mitochondria. We expressed the GFPNb in epidermis, cortex and lateral root cap as we expected ubiquitous constitutive expression to be lethal for the plant. Moreover, the epidermis and cortex cell files are easily accessible for imaging purposes. Proteins fused to this mitochondrial targeting signal colocalized with MitoTracker in transient *N. benthamiana* experiments (Winkler et al., 2021) and also here, we found our GFPNb to colocalize with the mt-rk mitochondrial marker in small punctae (Nelson et al., 2007). We also observed larger aggregates of signal, which we assume to be clustered dysfunctional mitochondria, similar to what we observed with our knocksideways strategy (Winkler et al., 2021). Constitutively decorating mitochondria with a GFPNb construct in the root epidermis and cortex cell files, therefore, might affect mitochondrial functionality without however causing a severe penalty on overall plant growth. The GFPNb system was capable of delocalizing TML-GFP and this caused the appearance of strongly fluorescent GFP-positive aggregations. Detailed inspection revealed however that our approach was insufficient to remove all TML from the PM. Compared to the control cells, sequestration of TML-GFP led to an overall reduction in signal intensity at the endocytic foci, as well as a reduction in overall density of endocytic foci when this was calculated as the amount of maxima that could be identified within a region of interest. The observed reduction of TML at the PM correlated with a significant reduction in endocytic tracer uptake, a proxy for reduced endocytosis.

The absence of major developmental defects, observed when TML-GFP was delocalized in the GFPNb lines can be explained by the fact that not all complex was delocalized as well as by the limited-expression domain of the PIN2prom. The increased root growth observed for the TML-Nb2 line is likely not linked to the delocalization of TML-GFP as we did not observe this in the TML-Nb1 line. An alternative explanation could be that the growth-promoting effect might possibly be a consequence of a positional effect of the insert. Inducible overexpression of AUXILIN-LIKE1/2 results in complete seedling growth arrest with drastic effects on cell morphology (Adamowski et al., 2018). The same holds true for inducible expression of dominant-negative clathrin HUB and DRP1A (Kitakura et al., 2011; Yoshinari et al., 2016). Furthermore, estradiol-inducible TPLATE and TML knockdown lines are noticeably shorter and show bulging cells (Gadeyne et al., 2014). As we did not observe cellular effects in epidermal or cortical cell files, we conclude that our approach lacked the required strength to block endocytosis, but only reduced it.

Recent results suggest that plant cells very likely contain a feedback loop controlling TPC expression, as carbon starved plants contained roughly the same amount of full-length TPLATE-GFP, next to an extensive amount of TPLATE-GFP degradation products (Wang et al., 2019). In case plant cells make more TPC upon depleting the complex at the PM, DeGradFP could provide a viable solution to this problem (Baudisch et al., 2018; Ma et al., 2019; Sorge et al., 2021). By applying this method in GFP-complemented *tml-1(−/−)* mutants, newly synthesized TML-GFP would be broken down immediately, preventing to achieve functional levels of TPC at the PM. Stronger or inducible promotors and/or the use of a different targeting location might also increase the delocalization capacity. To avoid lethality due to ubiquitous sequestration, engineered anti-GFP nanobodies, whose affinity can be controlled by small molecules, could also be used (Farrants et al., 2020).

Untangling the function of TPC and AP-2 in CME at the PM requires tools that allow interfering specifically with the functionality of both complexes. Our nanobody-dependent approach targeting TPC *via* TML resulted in the co-delocalization of one of the large subunits of AP-2, indicating that we likely are not only targeting TPC, but also AP-2 function. Whether a complementary approach, by delocalizing AP-2, using AP2S or AP2M in their respective complemented mutant backgrounds, would also delocalize TPC is something that would be worth trying. Furthermore, as AP2S and AP2M subunits are still recruited in *ap2m* and *ap2s* single mutant backgrounds (Wang et al., 2016), AP-2 in plants might also function as hemicomplexes similar to what is reported in *C. elegans* (Gu et al., 2013). Single mutants therefore might not reflect functional null *ap2* mutants and a similar approach as performed here might also provide tools to inactivate AP-2 as a whole, which can be highly complementary to working with the single subunit mutants.

In conclusion, the data presented here is a first step toward the development of specific tools, which are required to help us understand the functions of AP-2 and TPC. In the long-term, this will generate insight into endocytosis at the mechanistic level and this will bring us closer to being able to modulate CME-dependent processes, and thereby modulating plant development, nutrient uptake as well as defense responses to our benefit.

## Data Availability Statement

The raw data supporting the conclusions of this article will be made available by the authors, without undue reservation.

## Author Contributions

JW, ADM, EM, and PG designed and performed experiments. DVD designed experiments and wrote the initial draft together with ADM and JW. VS performed root growth assay statistical analysis. All authors contributed to the article and approved the submitted version.

### Conflict of Interest

The authors declare that the research was conducted in the absence of any commercial or financial relationships that could be construed as a potential conflict of interest.
